# Impact of Pre-Sleep Visual Media Exposure on Dreams: A Scoping Review

**DOI:** 10.3390/brainsci14070662

**Published:** 2024-06-29

**Authors:** Ajar Diushekeeva, Santiago Hidalgo, Antonio Zadra

**Affiliations:** 1 Department of Psychology, Université de Montréal, C.P. 6128, Succursale Centre-ville, Montréal, QC H3C 3J7, Canada; ajar.diushekeeva@umontreal.ca; 2 Department of Art History and Cinematographic Studies, Université de Montréal, C.P. 6128, Succursale Centre-ville, Montréal, QC H3C 3J7, Canada; santiago.hidalgo@umontreal.ca; 3 Center for Advanced Research in Sleep Medicine, CIUSSS-NÎM—Hôpital du Sacré-Cœur de Montréal, 5400 Gouin Blvd Ouest, Montréal, QC H4J 1C5, Canada

**Keywords:** dreams, dreaming, media consumption, REM sleep, NREM sleep, dream incorporation, dream content

## Abstract

A body of experimental research has aimed to investigate processes underlying dream formation by examining the effects of a range of pre-sleep stimuli and events on subsequent dream content. Given its ever-growing presence and salience in people’s everyday lives, pre-sleep media consumption stands out as a key variable that could influence people’s dreams. We conducted a scoping review to evaluate the experimental evidence of the effects of pre-sleep exposure to visual media on dream content. A systematic search on PubMed, PsycInfo, and Web of Science using terms related to moving visual media and dreams yielded 29 studies meeting the inclusion criteria. Overall, we found modest yet varied effects of pre-sleep exposure to visual media on dream content, with rates of stimulus-related incorporation ranging from 3% to 43% for REM dream reports, 4% to 30% for NREM sleep mentation reports, and between 11% and 35% for home dream reports. Our review highlights the large methodological heterogeneity and gaps across studies, the general difficulty in influencing dream content using pre-sleep exposure to visual media, and suggests promising venues for future research to advance our understanding of how and why digital media may impact people’s dreams.

## 1. Introduction

Dream formation draws upon a rich tapestry of sources encompassing semantic, episodic, and autobiographical memories, from current concerns to recent and remote experiences, psychological history, and sociocultural background [1,2,3]. Much scholarly attention in the field of dream research has been devoted to understanding how certain experiences, such as events from the preceding day, impact dreams [4,5,6]. To better understand how various events impact people’s dreams and uncover the underlying processes and potential functions of dreaming, researchers have attempted to influence dream contents by systematically manipulating pre-sleep and during-sleep experiences.

Within this line of research, different modalities of sensory stimulations have been administered during sleep (see [4,7,8] for reviews). These include somatosensory stimuli such as water sprayed on the skin [9], pressure stimuli [10], thermal stimuli [11], and electrical stimuli [12]; olfactory stimuli like pleasant and unpleasant odors [13]; visual stimuli like flashes of light [9]; auditory stimuli such as tones [14]; verbal stimuli such as tape recordings of persons’ names [15], and neutral versus meaningful words [16]; and even transcranial direct current stimulation [17].

Similarly, a range of stimuli and experiences have been experimentally presented prior to people’s sleep (see [4,18] for reviews), including group therapy sessions [19], periods of free association [20], hypnotic suggestions [21], studying [22], mental task performance [23], physical activity [22], fluid deprivation [24], food deprivation [11], social isolation [25,26], visual inverting prisms [27], and film viewing [28].

In addition to during-sleep sensory stimulation and pre-sleep priming, recent approaches to “dream engineering” (i.e., using techniques and technologies to manipulate dreams) have been employed, including dream incubation (i.e., pre-sleep rehearsal of content aiming to incubate desired dream features) and targeted memory reactivation (i.e., pairing pre-sleep and within-sleep stimulation to elicit reactivation of specific content; see [1] for a review). A substantial body of research has thus examined whether external stimuli can consistently alter sleep mentation, while deploying dream-incorporated stimuli as an experimental paradigm for investigating the mechanisms of dream production [4,8].

Media use is a particularly intriguing type of pre-sleep event; all the more so given the now-pervasive presence of digital media in our daily lives, including during our bedtime routines. For instance, one representative survey found that more than half of Canadians report that checking their smartphones is the last thing they do before going to sleep [29]. There is also a growing body of evidence suggesting that virtual experiences during our waking life (e.g., video game play; [30]) infiltrate our dreams, aligning with the continuity hypothesis of dreaming, which posits that our dreams embody and reflect our waking concerns and experiences [31,32,33].

Several correlational studies have noted associations between various measures of dream content and preceding media use, including overall media consumption, level of daytime exposure, and exposure prior to sleep onset. For example, in one study that sampled 3167 children aged 6–18 years, 74% of children reported that their dreams reflected what they had viewed on television or in films [34], while in another study, 53% of participants of a wide age range reported experiencing television-related dreams [35]. In pediatric populations, watching television and playing computer games has been linked with the frequency of TV-related dreams [36] as well as with unpleasant and pleasant dreams featuring TV content [37,38]. Another study found an association between the consumption of violent and sexual media before bedtime and the occurrence of violent and sexual dreams [39]. Moreover, playing video games has been shown to be associated with the incorporation of video game content into dreams [40], self-rated violence in dreams [41], and lucid dreaming [42]. Overall, social media use, especially engagement, has been linked to the prevalence of social media dreams [35,43]. Interestingly, children may be more susceptible to the effects of media use, as many report that watching TV has a more pronounced effect on their dreams than other daytime experiences [34]. In contrast, adolescents report that both watching TV and engaging in other daily activities impact their dreams in equal measure, and adults declare that daily activities have a greater influence on their dreams compared to watching TV [44]. While this correlational research provides valuable observations on the relationship between visual media use and dream content, it does not allow for causal inferences. Moreover, self-reported retrospective estimates of such effects may capture participants’ beliefs about how media manifests in their dream life, rather than measuring the actual impact of media exposure on dreaming.

Turning to experimental research, a number of studies have investigated how filmic stimuli, mainly stressful, arousing, and neutral films, influence various measures of dream content. These studies have yielded varying degrees of consistency in terms of how and to what extent these stimuli are incorporated into people’s dreams. Many of these experiments were conducted between the 1960s and 1990s, and as a result, the kinds of media stimuli employed in these studies may not accurately reflect the current media landscape. Some newer research has utilized interactive visual stimuli, including the computer game *Tetris* and an alpine ski visuomotor simulator game, demonstrating a significant degree of direct incorporation of game-related imagery into sleep-onset mentation while providing insights into the role of sleep in memory consolidation and learning [45,46,47]. Since then, a few other studies have examined the effects of immersive and interactive visual stimuli, such as video games and virtual reality, on dreams during REM sleep, the sleep stage most consistently and robustly associated with vivid, emotionally salient, and narratively driven dreams [48].

As digital media continues to shape and transform every aspect of our waking lives, it would be surprising if these stimuli did not exert an influence on our dream lives. Moving visual media, including activities such as streaming movies or shows, watching short-form videos, and gaming, represents one of the most pervasive and immersive forms of modern media consumption. Given its ubiquity facilitated by the use of smartphones, moving visual media emerges as a compelling factor with the potential to shape our dream experiences. To help unravel the intricate relationships between various kinds of pre-sleep media uses and subsequent dream content, we undertook a comprehensive examination of the existing literature. More specifically, we conducted a scoping review to systematically chart, describe, and synthesize the experimental research on the effects of exposure to moving visual media prior to sleep on subsequent dream content.

## 2. Materials and Methods

We adopted Arksey and O’Malley’s [49] methodological framework for conducting scoping reviews, as well as consulting the updated methodological recommendations outlined by the Joanna Briggs Institute [50]. The review process encompassed five stages, which are described below.

### 2.1. Stage 1: Identifying the Research Question(s)

This scoping review aimed to summarize and examine the experimental evidence regarding the impact of visual media stimulus exposure prior to sleep on ensuing dream content and features. How have visual media stimuli (e.g., in terms of methodologies) been utilized to influence dream content? What were the overarching objectives behind such endeavors, and which theoretical perspectives have informed this topic? What outcomes (e.g., incorporation rates of stimuli into dreams) have been reported? What approaches seem to be most efficacious or promising?

### 2.2. Stage 2: Identifying Relevant Studies

To identify eligible studies, we performed a systematic search of three databases: PubMed, APA PsycNet (PsycInfo and PsycArticles), and Web of Science Core Collection. The initial search was conducted in June 2021, followed by a subsequent search update across all databases in July 2023. The initial search strategy was developed within PubMed, using Medical Subject Headings (MeSH) terms and keywords such as “film”, “movie”, “video”, “television”, or “virtual reality”. This set of search terms was then combined using the Boolean operator “AND” to a second set of search terms focusing on “dreams” or “sleep mentation”. The search strategy formulated in PubMed was then adapted to suit the syntax of other databases. The search strategy encompassed peer-reviewed articles from any time period but limited itself to those published in English. A detailed search strategy is provided in Appendix A. Additionally, we manually checked the reference lists of selected publications (i.e., backward citation tracking) as well as their citing references via Google Scholar (i.e., forward citation tracking) to identify any potentially relevant articles that might not have been captured by the database searches.

### 2.3. Stage 3: Study Selection: Inclusion and Exclusion Criteria

**Figure 1:**
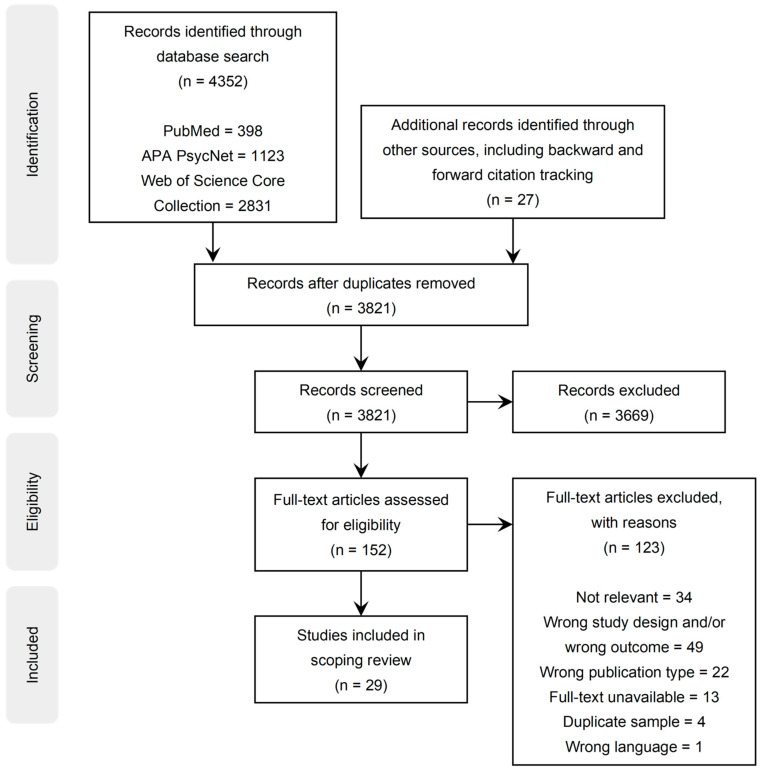
Preferred Reporting Items for Systematic Reviews and Meta-Analyses extension for Scoping Reviews (PRISMA-ScR) flow chart.

All the retrieved records were imported into a reference management software, *Zotero* 5.0, where duplicates in the library were detected and eliminated. We proceeded with screening the titles and abstracts of these records and excluded those extraneous to our topic. A total of 152 articles were retained for full-text review. The inclusion criteria covered experimental studies that investigated the effects of pre-sleep exposure to moving visual stimuli, such as films, videos, video games, and virtual reality, on subsequent dreaming, regardless of whether this constituted the primary focus of the study. Studies sampling adult, adolescent, and pediatric populations were considered eligible. The following exclusion criteria were applied: studies that were not experimental, lacked controlled exposure to moving visual media during wakefulness prior to sleep, or involved exposure to *static* visual stimuli (e.g., photographs), as our review focused on exposure to *moving* visual media since this form of media is most intimately tied to the question of how modern pre-sleep media consumption (e.g., TV shows, streaming services, etc.) may influence people’s dreams. We also excluded studies that did not assess dream content as an outcome or failed to report sufficient dream-related results. Only published articles were included; conference proceedings, abstracts, and dissertations were excluded, though they are briefly mentioned in the discussion if relevant. Studies employing duplicate participant samples were also excluded, while only the most relevant study for each distinct sample was retained to avoid redundancy and ensure the inclusion of unique data. A total of 29 articles satisfying the criteria were selected for data extraction. Figure 1 provides a representation of the screening process, in accordance with the Preferred Reporting Items for Systematic Review and Meta-Analysis Extension for Scoping Reviews (PRISMA-ScR) framework [51].

### 2.4. Stage 4: Charting the Data

The data charting process involved extracting the following information from the selected studies: bibliometric characteristics (author(s), year of publication, title, location); methodological data (sample size and characteristics, study design, pre-sleep stimulus exposure, measurement(s) of additional variables, study aims and hypotheses); outcomes (findings pertaining to dream content and proportion of stimulus-incorporation into dreams).

### 2.5. Stage 5: Collating, Summarizing, and Reporting Results

Some of the extracted data, including study characteristics and stimulus incorporation rates, were organized and summarized in tabular form. Table 1 presents all the included studies, which are organized into three groups: those conducted in a laboratory, those carried out at home, and those conducted in both laboratory and home settings. Further synthesis is provided in narrative form under the following themes: study and sample characteristics, theoretical frameworks, methodological characteristics, and relevant outcomes.

## 3. Results

Database searching yielded a total of 4352 records. After the removal of duplicates, 3794 records were screened based on their titles and abstracts. Among these, 125 records were retained, while an additional 27 records were identified through manual searching (i.e., backward and forward citation tracking), leading to a total of 152 records that underwent full-text screening. The reasons for exclusion are provided in Figure 1. The final selection consisted of 29 studies fulfilling the inclusion criteria.

### 3.1. Study and Sample Characteristics

The studies were published between 1964 and 2021. The majority of the studies were conducted in the United States (n = 12) [28,45,46,52,53,54,55,56,60,61,63,66] and Canada (n = 9) [57,59,64,65,68,70,72,73,76], while the rest were conducted in Germany (n = 3) [58,62,67], Australia (n = 2) [69,71], Belgium (n = 1) [47], The Netherlands (n = 1) [74], and France (n = 1) [75]. The sample sizes ranged between 3 and 137 participants, totaling 1193 participants across all the studies (51.2% male, 48.7% female). Gender information was missing in one study [45]. The age range spanned from 6 to 62 years, with a weighted average age of 21 years computed from the mean age data provided by seventeen studies. Four studies did not disclose any age-related information, while eight studies only reported age ranges. A significant portion of studies recruited university students (18/29; 62%), while only two studies investigated pediatric populations [53,55]. Nearly all the studies focused on healthy populations, with only one notable exception that recruited patients with medial temporal lobe damage in addition to healthy participants [45]. A few studies included distinctive populations, including night shift workers, *Tetris* experts, frequent video game players, and meditators [45,52,56,65,70,73]. Several studies lacked information regarding screening criteria. Table 1 outlines all the included publications along with their sample characteristics.

### 3.2. Theoretical Frameworks

The reviewed studies adopted a broad range of theoretical frameworks. Certain studies primarily sought to assess the effects of stimuli on the content of dreams, whereas others made use of this experimental paradigm to address other research questions while tangentially observing such effects. The first wave of studies addressing the effects of various stimuli on dream content, conducted in the 1960s and 1970s, broadly aimed to explore how experimentally controlled pre-sleep stimuli could be utilized to influence the dream formation process [28,53]. Some also aimed to investigate how emotionally charged and stressful experiences during wakefulness were integrated and transformed within subsequent dreams [52,54,56], while one study specifically endeavored to examine the effects of violent media on children’s dreams [55].

Several ensuing studies focused on the potential adaptive functions of dreaming. For instance, De Koninck and Koulack [57], as well as Lauer and colleagues [58], examined two contrasting hypotheses regarding the functions of dreams: the mastery hypothesis, which suggests that dreaming offers a means of gaining mastery over stressors, and the compensation hypothesis, which argues that dreaming plays a compensatory role by introducing content that is lacking from our waking life. In a similar way, Davidson and colleagues [69,71] set out to test Hartmann’s connectionist theory [77], which postulates that dreaming helps us process emotional concerns by forming new connections. One study in particular drew on Revonsuo’s threat simulation theory of dreaming [78] to examine how video game play may protect against nightmares [73]. Many contemporary studies (12/29; 41%) aimed to test the idea that dreaming contributes to sleep-dependent memory consolidation, usually by examining whether dreaming about a pre-sleep learning task is associated with improved post-sleep memory performance for that task (see [79] for a meta-analysis).

Finally, other studies examined specific features of dream formation without necessarily investigating the functions of dreaming. For instance, two studies focused on the temporal patterns of incorporation of memory elements into dreams [68,72], one evaluated how expressing feelings about pre-sleep events influenced their incorporation into dreams [59], another examined how video game immersion affected incorporation [70], while two others used virtual reality tasks to induce lucid dreaming and flying dreams [74,76].

### 3.3. Methodological Characteristics

#### 3.3.1. Types of Pre-Sleep Stimuli

Just under half of the studies (13/29; 45%) solely employed filmic stimuli, often comparing stressful films with neutral ones. One study among these described using a soundless film clip but did not provide a rationale for omitting the sound [67]. The use of film sequences was most common in studies from the 1960s to the 1990s, with only one recent study exclusively employing a film sequence [67].

Another group of studies utilized either video games, virtual tasks, or virtual reality (VR) tasks as stimuli (16/29; 55%). Two studies employed the classic puzzle video game *Tetris* [45,47], and four utilized virtual maze tasks in which the participants navigated a three-dimensional environment to solve a maze [60,61,63,66]. Another three studies employed visuomotor video games requiring the participants to engage in whole-body movements, with two of those choosing the *Wii Fit* video game [62,65] and another opting for an alpine skiing video game [46]. One study selected a first-person action–adventure video game on a *PlayStation 3* console, implementing four conditions varying in fidelity (i.e., high immersion versus low immersion) and interactivity (playing versus watching only) [70]. Four studies used VR tasks, including a VR maze task [72], a VR spatial memory task [75], a VR flying task [76], and VR video games developed for lucid dreaming training [74]. Solomonova and colleagues’ VR maze task resembled the virtual maze tasks mentioned above, but it incorporated VR goggles, thereby making it even more immersive [72]. Lastly, two studies employed more than one type of task. In one of these studies, a virtual maze task was compared with a visuomotor tennis video game on the Wii console [64], while the other compared three conditions: a first-person shooter combat video game, a non-combat creative video game, and a non-video academic searching task on the computer, with all conditions including exposure to a fearful movie sequence [73]. Table 1 offers a summary of the types of stimuli employed in each study included in the present review.

#### 3.3.2. Stimulus Exposure

In twenty-seven of the twenty-nine studies (93%), the participants were exposed to stimuli in a laboratory setting. In two studies, the participants engaged with the stimuli at home, where they viewed the videos on a DVD [69,71]. Usually, the participants were exposed to stimuli for durations ranging from 5 to 90 min over the course of one to three sessions. However, there were three exceptions where the participants experienced significantly longer periods of exposure. In two studies involving *Tetris*, the participants played the game for 6 to 7 h over the course of three consecutive days [45,47]. In a third study, the participants underwent VR lucid dreaming training for a total of 9 h, spread across twelve sessions over four weeks [74]. Three studies did not specify the duration of stimulus exposure [52,55,75].

#### 3.3.3. Sleep Context

In two-thirds of the studies (19/29; 66%), the participants slept in a laboratory environment. Of these nineteen studies, thirteen involved overnight sleep periods, four involved daytime naps [47,60,64,65], and two involved full daytime sleep sessions in male night workers [52,56]. Conversely, the participants slept in the comfort of their own homes in nine of the twenty-nine studies (31%). Notably, Picard-Deland and colleagues’ study featured both laboratory daytime naps and home sleep [76]. Table 1 categorizes studies according to three sleep settings, namely laboratory-based studies, home-based studies, and concomitant lab- and home-based studies.

#### 3.3.4. Dream Report Collection

Almost all of the laboratory-based studies used standard electroencephalography (EEG) to monitor sleep architecture and collect dream reports following scheduled awakenings. An exception was noted in the study by Stickgold and colleagues, where the Nightcap, a portable sleep monitoring system, was employed in a laboratory setting [45]. The home-based studies required the participants to maintain dream logs at home over a specified period of time, typically ranging from 1 to 14 nights. Exceptionally, one study required the participants to keep a home dream diary over a period of 6 weeks [74]. Only one study conducted in a home setting relied on a sleep monitoring device, Nightcap, to induce awakenings during specific sleep stages [46]. Finally, Picard-Deland and colleagues supplemented laboratory-collected dream reports following scheduled awakenings with home dream logs [76].

#### 3.3.5. Sleep Stages

Out of the twenty-one studies that monitored sleep stages and induced experimental awakenings, nine focused exclusively on REM sleep, five centered solely on NREM sleep (three targeted N1 and N2, and two targeted N1 alone), and seven examined both REM and NREM sleep (see Table 1 for details on the sleep stages focused upon in each of the studies).

#### 3.3.6. Dream Content Analysis

A majority of the studies (23/29; 79%) assessed the incorporation of stimuli into subsequent dreams. However, seven of these twenty-three studies either did not report global incorporation rates or failed to provide sufficient data to determine them [54,57,59,64,67,70,71]. Table 1 presents incorporations rates (expressed in percentages) for the remaining sixteen studies which either explicitly reported this information or included sufficient data to allow for their calculation. Across the studies, incorporation was typically scored by two to three blind judges. However, two of the studies did not specify whether their judges were blind [59,62], while four of the studies did not disclose any information regarding the involvement of judges [45,54,60,64].

Stimulus incorporation was often measured in a categorical fashion by classifying dreams based on the presence or absence of stimuli-related elements within the dream reports. Some studies categorized incorporations into subtypes. For instance, seven studies distinguished between direct or indirect incorporations [46,47,61,63,65,66,72], two studies differentiated between “certain” and “uncertain” incorporations [28,53], one differentiated between “sure” and “probable” incorporations [58], another between “literal”, “closely associated”, and “distantly associated” incorporations [71], and yet another between “primary” and “secondary” incorporated elements [70]. Three studies further classified incorporations according to the modality of their representation in the dream reports, distinguishing between thoughts, imagery, and visual, kinesthetic, and auditory sensory classes [45,46,47]. Two studies notably compared external ratings with self-reported ratings of incorporations [62,70].

In contrast, other researchers opted for continuous measures of the degree of incorporation into dream content. For instance, Kuiken and colleagues computed the ratios of the number of incorporations relative to the number of opportunities for incorporation [59], Powell and colleagues used a 10-point incorporation likelihood scale [68], while Gackenbach and colleagues developed their own analysis tool to detect video game elements within dreams [70]. For their part, Fogel and colleagues computed semantic similarity scores between wake task reports and subsequent dream reports [64]. Finally, Klepel and Schredl designed a film–dream similarity scale [67], while Ribeiro and colleagues employed a word matching strategy to derive an incorporation probability score [75].

In addition, a range of scoring scales and systems were used across thirteen of the twenty-nine studies (45%) to analyze diverse aspects of dream content aside from stimulus incorporation. These included scales devised by the research teams themselves (n = 5) [53,55,57,58,65], scales adapted from the Thematic Apperception Test (n = 1) [28], the Hall and Van de Castle [80] coding system (n = 3) [54,57,70], Gottschalk, Gleser, and Springer’s [81] anxiety and hostility scales (n = 2) [55,56], Hartmann’s [82] central imagery scoring system (n = 2) [69,71], Schredl’s [83] scoring scales (n = 1) [62], and Revonsuo and Valli’s [84] dream threat scale (n = 1) [73]. The rating process usually involved two to four blind judges, although two studies did not specify the blindness of their raters [62,73], two relied on a single judge [56,70], while one study did not provide information about the use and blinding of the judges [54].

Beyond relying on incorporation ratings by external judges, over a third of the studies (11/29; 38%) asked the participants to provide self-ratings of their dreams [28,55,58,59,60,62,70,71,73,74,76]. Finally, one study did not quantify dream content or apply any statistical treatment, instead adopting a qualitative psychoanalytic method to examine the presumed symbolic transformation of filmic stimuli in dream reports [52].

### 3.4. Narrative Overview of Outcomes

#### 3.4.1. Laboratory-Based Studies

In the 1960s and 1970s, a series of studies investigated the effects of stressful, arousing, and neutral films on dream content. The first study of this kind, by Foulkes and Rechtschaffen, found that when compared to the effects of a comedic Western film, a violent Western film produced REM dream reports that were longer and more imaginative, as rated by external judges, as well as more vivid and emotional, as rated by the participants themselves [28]. The violent film, however, did not result in dreams that were more unpleasant or violent in comparison to the nonviolent film. The authors reported a net incorporation rate of 5%, with clear film elements appearing in 5.5% of the REM reports and 3.8% of the NREM reports. Contrastingly, another study by Foulkes and colleagues with an incorporation rate of 8% revealed that a baseball film elicited more imaginative, hostile, guilty, and well-recalled dream reports in boys than did a violent Western film [53]. While adult participants preferred the comedic film in the former study, the boys in the Foulkes et al. study showed greater interest in the violent film [53]. However, a subsequent study of boys by Foulkes and colleagues found no significant relationship between hostility or other dream variables and the type of film viewed, whether violent or nonviolent [55]. They observed that increased attentional involvement with the films led to greater dream hostility, but they refrained from drawing conclusions regarding the link between interest or involvement levels and dream intensity or hostility.

A study by Witkin and Lewis, which presented qualitative results based on data from only three participants, observed that films about birth and subincision led to dreams “filled with obvious sexual symbolism”, while neutral films did not [52]. The researchers also found a higher frequency of “forgotten dreams” (i.e., awakenings with no dream recall) following arousing films compared to neutral ones. In a study by Cartwright and colleagues, erotic films resulted in more one-character dreams and less heterosexuality, with an ensuing rise in two-character dreams on the last night of five consecutive experimental nights [54]. While no direct film incorporation was observed, the authors reported frequent symbolic representations of sexual material. In another study, birth and subincision films provoked increased dream anxiety and decreased social affection compared to neutral films, particularly among the participants who reported more waking anxiety after viewing the films [56]. Finally, De Koninck and Koulack found no significant effects of stressful films depicting workshop accidents on dream content [57].

A subsequent laboratory-based study found that stressful films depicting a cruel massacre and the brutality of the prison system elicited more anxious and aggressive dreams during the first REM period compared to a neutral film [58]. Self-rated dreamer participation was higher following exposure to the stressful films than the neutral film. Overall, 37.5% of the dream reports contained clear incorporations of stressful films, with 33% of the initial REM reports and 43% of the final REM reports incorporating film elements. Close to 55% of the participants integrated film elements into their dreams.

In another study, emotionally engaging films addressing themes of mortality and mental health were shown to participants in two conditions: the “feeling expression” condition, in which the participants were prompted to reflect on the film segment most significant to them, and the “no feeling expression” condition, in which the participants were not provided with the same opportunity to express their feelings [59]. Dreams stemming from the feeling expression condition were found to be more affectively similar to the films but less likely to incorporate film narrative elements, particularly actions, compared to the no feeling expression condition. Dreams from the feeling expression condition also exhibited more self-reference than those from the no feeling expression condition.

More recent studies in the 2000s and 2010s shifted towards the use of video games and virtual tasks as stimuli. Stickgold and colleagues reported that 7% of sleep-onset mentation reports produced by amnesic and normal participants alike incorporated explicit *Tetris* elements that closely mirrored the visual imagery encountered during awake gameplay, with 63% of all the participants experiencing *Tetris*-related hypnagogic imagery [45]. Mentation reports containing *Tetris*-related thoughts were absent in amnesiacs who only reported *Tetris* imagery, while non-amnesic *Tetris* novices reported both. Interestingly, *Tetris* imagery in non-amnesiacs increased across nights, with 90% of imagery reports occurring on the second night, whereas *Tetris* thoughts appeared more frequently on the first night. The incidence of *Tetris* imagery dropped by two-fold over a two-minute period at sleep-onset, while *Tetris* thoughts remained constant during this timeframe. In Kussé and colleagues’ study, a remarkable 81% of the participants incorporated *Tetris* elements into sleep mentation [47]. *Tetris* content was incorporated into 10% of the mentation reports, consistently across three days of testing. Among these incorporations, one-third were direct incorporations and two-thirds were indirect. While 11.2% of N1 reports and 6.5% of N2 reports in this study contained *Tetris* elements, this difference did not reach statistical significance. However, there was a significant variation in the occurrence of *Tetris*-related reports across sensory classes, with visual imagery being the most prevalent.

Wamsley and colleagues reported that 8% of the participants produced sleep mentation unambiguously related to a virtual maze task, with 6% of the participants experiencing them during N1 and 2% during N2 [60]. In a subset of participants instructed to report at the end of an uninterrupted sleep opportunity whether they had experienced maze-related sleep mentation, 55% answered affirmatively. In another study, 37% of the participants incorporated content related to a virtual maze task [61]. About 9% of these reports were maze-related, with almost 4% directly associated with the task and 5% solely indirectly associated. The occurrence of maze-related reports was consistent across N1, N2, REM, and morning awakenings. Interestingly, introducing a monetary reward and performance feedback to the task did not affect the incorporation levels in the dream reports. In another study by Wamsley and colleagues, the incorporation rate of a virtual maze task proved to be much lower—1.9% of all reports—with 12% of the participants incorporating maze-related content [63]. A test expectation manipulation (i.e., whether the participants were informed about an upcoming memory test) did not influence incorporation into dream reports. Wamsley and Stickgold observed that 6.4% of mentation reports directly incorporated a virtual maze task across all sleep stages [66]. Specifically, 7.1% of N1 reports, 2.1% of N2 reports, 12.5% of morning N2 reports, and 12.5% of morning REM reports contained maze-related elements. A total of 47% of the participants directly incorporated the maze task.

In a study by Fogel and colleagues that utilized both a virtual maze task and a visuomotor tennis video game for the Wii console, no significant difference was found in the degree of incorporation between the two stimuli [64]. The extent of incorporation into early versus late N1 sleep mentation also did not differ, but incorporation into early N1 mentation was positively associated with reasoning ability, while incorporation into late mentation was positively correlated with verbal ability. In another study involving a visuomotor video game for the Wii console and Wii Balance Board, researchers compared external ratings by judges with participants’ self-ratings of incorporation into REM dreams [62]. The external ratings indicated that 5.6% of the dream reports featured balance-related elements, with 15% of the participants judged as having incorporated balancing elements. Additionally, 47.2% of the dream reports were judged to reference the laboratory setting, and 85% of the participants were judged as having incorporated laboratory elements. The participants’ self-assessments revealed that almost 20% of the dream reports contained balance-related elements, with 54% of the participants reporting at least one balancing dream.

Another study featuring a visuomotor video game for the Wii console and Wii Balance Board showed that game elements were incorporated into 25% of REM reports, 18.8% of N2 reports, and 17.5% N1 reports, totaling 19.1% of all dream reports [65]. Conversely, laboratory references were found in 75% of REM reports, 37.5% of N2 reports, and 27.5% of N1 reports, accounting for 38.2% of all dream reports.

Finally, a study by Klepel and Schredl exposed participants to a short comedic film sequence and compared their subsequent dream reports with those of control participants who did not view the film [67]. Morning the dream reports obtained from the film-viewing group showed higher film–dream similarity compared to the control group, but no differences were found for the first REM dream reports collected at night. More film incorporations were observed in the morning dream reports than in the first REM reports.

In summary, laboratory-based studies conducted over several decades have yielded a broad range of outcomes, including mixed, and at times contradictory, results. The heterogenous nature of these outcomes is also reflected in the limited number of studies having quantified the proportion of dream reports showing evidence of stimulus-related incorporations, with percentages ranging from a low of 2% to a high of 38%.

#### 3.4.2. Home-Based Studies

In a study by Powell and colleagues, participants were shown a stressful film depicting a ceremonial buffalo slaughter and then asked to keep a dream diary for seven days to investigate the “dream-lag effect”, which refers to the delayed incorporation into dreams of waking events often experienced 6–8 days prior [68]. Nearly half (47.4%) of the participants were classified as high incorporators. A temporal U-shaped quadratic trend for film incorporations was observed across seven nights, specifically in high incorporators. Davidson and colleagues compared the effects of viewing 9/11 media coverage and a neutral psychology lecture on subsequent dreams [69]. They found that dreams following the 9/11 video showed higher levels of contextualizing imagery compared to those following the control video. Some dream elements directly reflected aspects of the 9/11 video, while others were thematically related. A measure of subjective stress after exposure to the 9/11 footage, rather than trait empathy, was associated with the presence of contextualizing imagery in dreams. Using the same stimuli, Davidson and Lynch reported that the 9/11 video produced dreams characterized by more intense central imagery and stronger negative emotions compared to the lecture video [71]. The 9/11 video also led to dreams with more literal, closely associated, and distantly associated 9/11 elements, as well as more thematic 9/11 imagery.

In another study, participants played a visuomotor alpine skiing video game, which was featured in 29.5% of mentation reports across all nights [46]. Of these reports, 23.6% were characterized by sensory imagery, while 6% contained game-related thoughts. Visual imagery predominated over other kinds of sensory modalities, although kinesthetic imagery was present in one-third of the reports. Direct and indirect incorporations accounted for 23.3% and 6.2% of game-related reports, respectively. Directly related imagery diminished with increasing time since sleep onset, as monitored by the Nightcap, suggesting that game-related imagery became more abstracted as time into sleep increased. Overall, incorporations were more common during awakenings scheduled earlier in the night, and only a small portion (1.28%) of the morning dream reports were game-related. Across three post-stimulus nights, there was a decline in the incorporations per participant. On the first night, 47% of the mentation reports produced by 65% of the participants were related to the video game. Remarkably, a small group of observers who simply watched others play the game showed similar rates of incorporation as active players. Self-reported task engagement did not seem to influence the incorporation rate.

In a study by Gackenbach and colleagues, participants played an action–adventure video game under one of four conditions varying in fidelity (i.e., wearing immersive goggles versus no goggles) and interactivity (playing the game versus watching a recorded gaming session) levels [70]. The high fidelity–high interactivity condition yielded the highest rate of incorporations according to both the judges’ ratings and the participants’ self-ratings, particularly on the first, sixth, and seventh nights of the self-reports. Curiously, the low interactivity–low fidelity condition produced the second most self-reported incorporations on these nights. The high fidelity–low interactivity condition showed the lowest primary incorporations and the highest laboratory incorporations, as scored by the judges. Self-rated emotional engagement during gameplay was not associated with incorporation into dreams. In another study by Flockhart and Gackenbach, high-end gamers and low-end gamers were all exposed to a fearful movie sequence and engaged in one of three activities: a combat video game, a creative video game, and a scholarly search task on the computer [73]. The findings indicated that high-end gamers who played the combat game experienced marginally fewer and less severe threats in their dreams, as evaluated by judges, compared to low-end gamers who played the same game. The high-end gamers also self-reported fewer bad dreams and less fear in their dreams after playing the combat game relative to the other two tasks. Conversely, the low-end gamers self-reported more bad dreams after playing the combat game compared to the other conditions. The participants who played the creative video game experienced higher levels of self-reported dream bizarreness.

In one among a series of contemporary studies utilizing VR tasks, Solomonova and colleagues observed that VR maze incorporations into dreams followed a distinct temporal pattern marked by a peak on days four and five over a ten-day period, while laboratory incorporations revealed a standard U-shaped quadratic pattern characterized by a day-residue effect and a delayed dream-lag effect [72]. A total of 11% of the dream reports featured VR incorporations on the first day post-VR, contrasting with 53% of the dreams referencing the laboratory. Overall, 61.5% of the participants incorporated the VR maze task into their dreams, with 12.5% of dreams collected per participant containing VR elements. Interestingly, VR and laboratory incorporations almost exclusively occurred in separate dreams. Dreams highest in VR incorporations were associated with a relatively more internal rather than external dream locus of control.

In a study by Ribeiro and colleagues, 35.3% of dream reports originating from 22% of participants incorporated items from a VR spatial memory task [75]. The researchers determined that the likelihood of observing the incorporation of these items into dreams of control compared to those who were not exposed to this VR task ranged from 0.18% to 4.23%. In another study, the participants partook in VR-assisted lucid dreaming training involving dream-like video games, amounting to 9 h of VR exposure over the course of four weeks [74]. While VR training led to increases in lucid dreaming compared to no training, it did not fare better than classical lucid dreaming training. There were no differences in dream lucidity or VR incorporation between the nights following VR training and those without preceding VR sessions.

In summary, home-based studies have found stimuli incorporation rates that are comparable to those observed under laboratory conditions, ranging between 11% and 35%. These home-based studies have also facilitated the collection of dream content data over longer periods of time, allowing researchers to investigate the temporal relationship between exposure to stimuli and their subsequent incorporation into dreams.

#### 3.4.3. Laboratory and Home-Based Studies

Finally, Picard-Deland and colleagues had participants complete a VR-flying task followed by a lab-based morning nap while also maintaining home dream journals for five days before and ten days after the VR exposure [76]. The authors found that the VR flying task resulted in an increase in the frequency of flying dreams, from 1.7% of the dream reports at baseline to 7.1% of the reports following VR exposure. Flying was also featured in 3.1% of the REM reports and 20% of the NREM reports collected during the laboratory nap session. A day-residue effect was observed on the first post-VR night, when the incidence of flying dreams peaked at 10.6% of the dream reports. Overall, 4.1% of the dream reports featured flying across the ten nights post-VR. In all, 4.4% of the participants experienced flying dreams in the laboratory, while 22.1% experienced them at home. Flying dreams were more likely to occur in the participants with previous flying and lucid dreaming experiences. The intensity of flying sensations within dreams was positively associated with the degree of immersion-proneness and cybersickness, but not with flying dream frequency.

## 4. Discussion

The goal of the present scoping review was to examine the experimental evidence pertaining to the effects of moving visual media on dream content. Overall, the results suggest that visual media exposure before sleep has the potential to alter dream content, but the degree and nature of this influence varies significantly. A synthesis of key outcomes and methodologies is presented below, and remaining questions and gaps in the literature are highlighted.

### 4.1. Effects of Moving Visual Media Stimuli on Dream Content

When considering the body of evidence as a whole, moving visual media stimuli have been shown to exert a moderate influence on dream content. Several studies have noted various changes in dreams following exposure to specific stimuli, including changes in dream recall, their length, emotional content (e.g., hostility, guilt, anxiety, fear), social interactions (e.g., affection, aggression, sexuality), characters, and other attributes (e.g., imaginativeness, vividness, bizarreness, threats, contextualizing imagery). However, these observed effects may not fully capture the extent of the impact of visual media stimuli on dream content, as the identification of such effects depends on each study’s specific objectives and hypotheses. In most later studies, researchers were not aiming to identify changes in dream features, so these may have gone unnoticed.

The rates of stimuli incorporation into dream reports appear to be rather modest, ranging from about 2% to 38% across all studies. The proportion of direct versus indirect incorporations also varies across studies, with direct ones typically occurring earlier in the night as well as earlier into the N1 sleep stage [46,47,61]. Most incorporations manifested as visual imagery [46,47]. This variability in incorporation rates could be attributed to several factors, including the heterogeneity in research methodologies, which complicates direct comparisons and prevents the delineation of more robust conclusions. A large number of methodological details differed from one study to the next, such as the duration, timing, and context of stimulus exposure, the protocol for collecting dream reports, and the assessment of incorporation and other dream-related variables. The next section highlights some of the factors that may have contributed to the observed variations in incorporation rates.

### 4.2. Methodological Differences and Other Factors Contributing to Variance in Outcomes

#### 4.2.1. Types of Stimuli

Hardly any studies have systematically compared different types of stimuli, making it challenging to determine which features of visual media may enhance the likelihood of incorporation into dreams. One notable exception is a study that cleverly manipulated the attributes of a video game by varying its levels of fidelity (i.e., immersive goggles versus no goggles) and interactivity (i.e., playing versus watching) [70]. The study demonstrated that highly immersive and interactive video gaming led to the highest rates of incorporation, as determined both by external judges’ evaluations and self-ratings. Similarly, another study, which was not included in the present review, as it has not been published, manipulated a virtual maze task’s level of interactivity and visual display [85]. It revealed that high interactivity (i.e., playing as opposed to merely viewing) resulted in more self-reported incorporations, while the type of visual display (VR goggles versus TV screen) did not significantly affect incorporation scores. In contrast, Wamsley and colleagues found that watching others play a visuomotor skiing video game led to similar incorporation rates (19% of observers’ dream reports) as actively playing it (24% of players’ dream reports), although the small sample size of observers (n = 3) warrants caution [46]. Additionally, a study by Fogel and colleagues compared a virtual maze task with a visuomotor tennis video game and observed comparable incorporation rates for both [64]. This suggests that the involvement of motor movements in gameplay, such as in the tennis game, do not necessarily enhance incorporation. However, because this study did not control or manipulate specific stimulus attributes, this conclusion remains speculative. Briefly put, drawing definitive conclusions about which types of stimuli are most prone to induce incorporations into dreams is difficult due to the lack of systematic comparisons. Levels of interactivity and immersion might play a role, but their relative importance remains unclear, given the inconsistent findings across the studies described above and the lack of a clearcut discrepancy in incorporation rates between studies employing filmic stimuli and those utilizing video games, virtual tasks, and VR.

#### 4.2.2. Stimulus Exposure

The impact of the duration of stimulus exposure, or stimulus “dose”, has not been investigated. Without controlled investigations varying exposure durations within the same experiment, it is difficult to hypothesize how duration might influence incorporation. Comparing incorporation rates across studies with extended periods of exposure to those with brief exposure reveals no straightforward relationship between the duration of exposure and the likelihood of incorporation. For example, playing *Tetris* for 6 to 7 h over three days does not appear to facilitate incorporation compared to engaging in virtual maze tasks or other video games for as little as seven minutes [45,47,65]. This aligns with previous research indicating that the amount of time spent on waking-life activities may not be the most reliable predictor of their incorporation into dreams [86].

#### 4.2.3. Perceived Salience and Engagement with Stimuli

The perceived salience of the stimuli and the level of involvement or engagement with it is another factor that might modulate its observed effects on dreams. Research indicates that emotionally charged and personally significant waking-life experiences are more likely to be integrated into dreams [87,88]. However, the studies included in the current review do not provide clear evidence in that regard. In two separate studies using similar filmic stimuli, opposite effects on dream content were observed in terms of the dreams’ imaginativeness and emotionality [28,53]. Foulkes and colleagues suggested that this divergence could be attributed to the differing levels of interest in one film stimuli over the other: adult participants in the former study favored a romantic comedy compared to a violent Western, whereas children in the latter study preferred the violent Western over a baseball film [53]. These complementary studies suggest that one’s sense of interest or investment in the stimuli may modulate their effects on dream content.

Another study by Foulkes and colleagues manipulated levels of attention allocated to films and observed that greater attention involvement resulted in more dream hostility [55]. Other studies that manipulated variables such as monetary reward, performance feedback, and test expectations in virtual maze tasks did not find that these factors moderated incorporation into dreams [61,63]. Although these variables might be expected to increase task salience and involvement, consequently enhancing incorporation, other factors such as high perceived task difficulty and negative emotional valence might have been at play [61]. Moreover, in some studies, self-rated task engagement and emotional engagement during gameplay were surprisingly not associated with incorporation rates [46,70].

While subjective salience and engagement might play a role in dream incorporation, their impact is not straightforward and may interact with other factors. Novelty is another factor that may impact the extent to which stimuli are incorporated into dreams, as some evidence suggests that novel and highly emotional experiences are preferentially incorporated into dreams compared to recurrent daily activities [87]. Despite this, no study has tried comparing the effects of familiar versus unfamiliar stimuli to explore the impact of novelty.

#### 4.2.4. Experimental Setting

A few studies conducted partially or fully in a laboratory have shown that laboratory-related elements are incorporated into dreams at substantially higher rates than intended stimuli, with incorporation rates of 53% versus 11% [72], 47.2% versus 5.6% [62], and 38.2% versus 19.1% [65]. These findings are consistent with other research indicating that over a third of dream reports collected at a laboratory reference the experimental setting [89,90]. Although the laboratory setting offers the advantage of closely monitoring sleep and collecting reports throughout the night, it may interfere with the incorporation of the intended stimuli [90]. In support of this, one study has shown that laboratory and stimulus incorporations rarely, if ever, appeared within the same dreams [72]. This interference likely arises because the experience of staying in the laboratory while undergoing polysomnography can be more salient, novel, and stressful to the participants than the stimuli they are exposed to, leading to a preferential incorporation of laboratory elements. This phenomenon may be more pronounced during the REM stage, which is most strongly associated with laboratory incorporations [90]. To mitigate this issue, experiments could include an adaptation night, allowing the participants to familiarize themselves with the environment before being exposed to the stimulus.

#### 4.2.5. Sleep Stages

The influence of sleep stages on the likelihood of incorporation into dreams appears to vary across different studies. Two studies have reported consistent rates of incorporation between N1 and N2 sleep, as well as across N1, N2, REM, and morning awakenings [47,61]. Another study has shown significantly greater incorporation in N2 compared to REM sleep [76]. Three additional studies, while monitoring the frequency of incorporations across different sleep stages, did not perform statistical comparisons to confirm whether incorporation rates differed meaningfully between stages. In one study, 6% of participants experienced incorporations during N1 compared to 2% during N2 [60]. In another study, 7.1% of N1 reports, 2.1% of N2 reports, 12.5% of morning N2 reports, and 12.5% of morning REM reports produced incorporations [66]. Yet another study showed incorporations in 25% of REM reports, 17.5% of N1 reports, and 18.8% of N2 reports [65]. These findings suggest that incorporations can occur during all stages of sleep, but it is not possible to determine which stage tends to outperform the others and under what circumstances.

#### 4.2.6. Time-of-Night

While the results from two studies seem to indicate that film incorporations are more common in final REM and morning dream reports compared to those collected during the first REM stage of the night [58,67], findings from another study contradict this trend [46]. The latter found that incorporations occurred more frequently for awakenings scheduled earlier in the night, with morning report incorporations proving to be less common. The rate of incorporation into N1 reports early in the night does not appear to differ from that of N1 reports later in the night, as reported in one study [64]. One study further observed that the occurrence of incorporated imagery seemed to decrease across two minutes into N1, while the incidence of incorporated thoughts remained constant [45]. Another study using the same stimulus found no changes in the incorporation rate over the same two-minute period since N1 onset, although it did not differentiate between imagery and thoughts [47]. Direct incorporations were shown to decrease as time since sleep onset increased in another study, suggesting a process of abstraction during the first minutes of sleep as well as through the night [46].

#### 4.2.7. Time Interval between Stimulus Exposure and Incorporation

A few studies have examined the temporal pattern of incorporation across post-stimulus nights, revealing varying findings. Some studies have identified a marked “day-residue effect”, wherein the incidence of incorporations peaked on the first night following exposure to the stimulus [46,68,76]. For instance, Wamsley and colleagues observed incorporations in a remarkable 47% of mentation reports on the first night, followed by a linear decline over the next two nights [46]. This is in line with other research indicating that daytime material tends to appear more frequently in dreams the following night [5].

A study by Powell and colleagues identified both a day-residue effect and a dream-lag effect, where a second peak in incorporations occurred on the sixth and seventh nights, demonstrating a bimodal U-shaped quadratic pattern across seven nights in total [68]. In contrast, another study observed no such dream-lag effect, despite showing a day-residue effect [76]. Curiously, Solomonova and colleagues observed a completely different temporal pattern, with incorporations peaking on days four and five over a ten-day period, speculating that the experience of staying in a laboratory may have competed with the VR experience [72]. Another study noticed a divergent distribution of incorporations for imagery versus thoughts: imagery peaked on the second night, while thoughts peaked on the first night [45]. However, these findings may be confounded by the fact that the participants were exposed to the stimulus across all three experimental nights.

#### 4.2.8. Dream Content Analysis and Scoring of Incorporations

Different scoring methods were used across studies or the assessment of dream-related variables and incorporations. Regarding content analysis in particular, some studies employed established analysis systems like the Hall and Van de Castle [80] scoring scales, while others relied on their own scoring systems. Varying criteria were also used to detect incorporations across studies, and this inconsistency could have impacted the proportion of reported incorporations. The variability in scoring standards, including the levels of criteria stringency, might have contributed to overestimates or underestimates of incorporation. A few studies also did not provide enough detail on what constitutes an “incorporation”, making it harder to confidently draw comparisons between studies. In some instances, it was also unclear whether evaluators were effectively blinded to the experimental conditions. Overall, studies could benefit from better standardization of scoring methods.

#### 4.2.9. External Ratings vs. Self-Ratings

Based on the limited number of studies that employed both external judges’ ratings and participants’ self-ratings of stimuli incorporations in dreams, it appears that self-assessments tend to estimate higher incorporation rates than those provided by judges. For example, Nefjodov and colleagues found that over half of their participants rated 20% of their dream reports as containing incorporations, whereas external ratings identified only a 5.6% incorporation rate [62]. Similarly, in another study, the judges determined that 8% of the participants produced reports with incorporations, while a much larger 55% of the participants self-reported such incorporations [60]. This disparity could be attributed to external raters potentially underestimating the quantity of incorporations due to a more restrictive scoring method. Conversely, participants’ ratings might be inflated due to demand characteristics. Each assessment method has its potential biases and limitations, so studies may benefit from using both concurrently [91].

#### 4.2.10. Trait and State Differences

Trait and state correlates that may be associated with incorporation amplitude remain largely unexplored. Nonetheless, a few findings are worth mentioning. Research by Fogel and colleagues indicates that different cognitive abilities may facilitate incorporation into dreams from the N1 stage [64]. Another study suggests that participants’ subjective stress response to a stimulus, rather than their empathetic disposition, may play a role in the way stimuli are reflected in dream contents [69]. One study involving high-end and low-end gamers suggests that the level of experience with a stimulus can mediate its impact on dream contents [73]. Another study found that prior dreaming experiences can increase the likelihood of incorporation of specific stimuli; participants with previous experiences of flying and lucid dreaming were more likely to report flying dreams [76]. Immersion-proneness predicted the intensity of flying sensations, showing that susceptibility to immersive media experiences can augment dream experiences [76]. This research highlights that factors such as cognitive abilities, emotional responses, and past experiences can potentially play a role in the incorporation of stimuli into dreams.

### 4.3. Considerations for Future Research

This section provides recommendations for future research directions in light of key methodological limitations in the field. First, the nature of stimuli, including their characteristics, represents an area of research that warrants clarification and further investigation. While existing studies have used various kinds of stimuli, their salience to participants may have been insufficient, and their impact varied considerably from each participant to the next. Future research should consider the roles of stimuli salience, engagement, and novelty as modulating factors that could potentially influence the occurrence of incorporations into dreams. For instance, no studies to date have allowed participants to select their own media stimuli, especially those personally relevant to them, in order to better investigate the impact of such subjective levels of stimuli salience on dream content and the extent of incorporation.

Further investigations are also needed to clarify the role of media attributes, including degree of immersion and interactivity. While some studies have demonstrated the importance of such attributes, it remains unclear under what circumstances they are most likely to exert their influence.

Future studies should also strive to better reflect current media use habits in their stimuli selection. Many experiments have used stimuli such as virtual maze tasks or disturbing film sequences, which do not represent activities that individuals typically engage in before sleep. To our knowledge, no studies have examined the effects of newer forms of media, such as social media platforms, which are expressly designed to retain user engagement and are used daily by many, particularly before bedtime. Aligning stimuli with up-to-date real-world media usage would ensure greater ecological validity of key experiments, especially those carried out in home settings.

Increasing the standardization of methods for assessing dream content and degree of incorporation is important for facilitating comparisons across studies. Researchers should continue exploring computational linguistics approaches [63] while comparing them with manual scoring methods to determine whether the former can better promote comparability and reproducibility across studies. Employing both external ratings and self-ratings as complementary methods may mitigate inherent biases and limitations in either approach. Moreover, extending the period of dream report collection to at least 14 days post-stimulus would be desirable, as this would allow researchers to capture potential incorporations that may arise later in time (i.e., the dream-lag effect) and to determine temporal patterns of incorporation.

There is also a greater need for laboratory-based studies to better document which sleep stages, and during what sleep cycles or periods of the night, are most likely to show evidence of dream incorporations of pre-sleep media stimuli. Lab studies should also aim to report incorporation rates separately for different sleep stages, as opposed to pooling them together, be it during regular overnight sleep assessments or during daytime naps. Similarly, home-based studies should, if possible, include portable sleep-monitoring devices to better track sleep stage awakenings as well as to carry out experimental awakenings in participants’ natural home environments.

Finally, future research should examine how trait-dependent interindividual differences modulate the relationship between visual media stimuli and dream content. Factors such as immersion and absorption proneness in mediated environments may well affect how media experiences are incorporated into dreams, yet our understanding of their contribution remains limited.

## 5. Conclusions

In conclusion, despite mixed results, the overall evidence compiled throughout this scoping review indicates that engaging with moving visual media (e.g., films, video games, virtual tasks) has moderate effects on dream content, including on the incorporation of media-related elements into dreams. The substantial variability in outcomes across studies highlights the need for further research to clarify the nature and magnitude of these effects and to better identify factors contributing to the observed variations. Notably, there exists a need for more targeted investigations in the field, as most of the reviewed studies were not explicitly designed to examine the impact of pre-sleep media exposure on dream content, but rather reported on these kinds of observations while perusing other research questions. Given the substantial role that various kinds of media now play in people’s daily lives, including the growing engagement with virtual environments, it is important to better understand how and why media comes to influence dream content. Moreover, such research efforts could lead to advances in media-assisted engineering of desired dream experiences and even the management of disordered dreaming [1]. Finally, such experimental protocols could provide valuable insights into the mechanisms through which various kinds of waking-life experiences are integrated into people’s dreams, thereby contributing to our understanding of the dream formation process and, ultimately, the possible function of dreams.

## Figures and Tables

**Table 1:** Study characteristics and stimulus incorporation rates of included studies (N = 29).

Study	Population	Stimulus	Sleep Stage			Incorporation Rate (% Content-Filled Dream Reports) ^a^	Incorporation Rate (% Participants) ^b^
			REM	NREM	N/A		
**Laboratory-Based Studies**							
Foulkes and Rechtschaffen (1964) [28]	24 students (13 m, 11 f)	Violent Western film Comedic Western film Duration: 30 min.	✓	✓		5% of dream reports 5.5% of REM reports 3.8% of NREM reports	N/S
Witkin and Lewis (1965) [52]	3 male nights workers	Birth film Subincision film Neutral travelogue film Suggestion session	✓			N/S	N/S
Foulkes et al. (1967) [53]	32 boys (6–12 y.o.)	Violent Western film Baseball documentary Duration: 10 min.	✓			8% of dream reports	N/S
Cartwright et al. (1969) [54]	10 male students	Two erotic films Duration: 10 min. each	✓			N/S	N/S
Foulkes et al. (1971) [55]	40 boys (10–12 y.o.)	Violent Western film Nonviolent Western film	✓			N/S	N/S
Goodenough et al. (1975) [56]	28 male night workers	Birth film Subincision film Neutral travelogue film Another neutral travelogue film Duration: 11 min. each	✓			N/S	N/S
De Koninck and Koulack (1975) [57]	24 male students	*It Didn’t Have to Happen*, film depicting workshop accidents Duration: 13 min.	✓			N/S	N/S
Lauer et al. (1987) [58]	11 men	Massacre film Prison film Neutral animal documentary Duration: 90 min.	✓			37.5% of dream reports 33.3% of initial REM reports 42.9% of final REM reports	54.5% of participants
Kuiken et al. (1990) [59]	12 students (6 m, 6 f)	*Where is Dead?*, film about death and grief *Dreamspeaker*, film about a boy who is committed to a mental institution	✓			N/S	N/S
Stickgold et al. (2000) [45]	27 volunteers (*Tetris* novices, *Tetris* experts, and amnesics)	*Tetris* Duration: 7 h in total		N1		7.4% of mentation reports by amnesics 7.2% of mentation reports by normals	63% of participants
Wamsley et al. (2010a) [60]	99 students (44 m, 55 f)	Virtual maze task Duration: 45 min.		N1 N2		N/S	8% of participants Among those, 6% during N1, 2% during N2 55% of participants in the questionnaire protocol group
Kussé et al. (2012) [47]	43 healthy volunteers (19 m, 24 f)	*Tetris* Duration: 6 h in total		N1 N2		10% of mentation reports Among those, 3.3% contained direct incorporations, 6.8% indirect incorporations 11.2% of N1 reports 6.5% of N2 reports	81% of participants
Stamm et al. (2014) [61]	65 healthy volunteers (37 m, 28 f)	Virtual maze task Duration: 35 min.	✓	N1 N2		8.5% of dream reports Among those, 3.7% contained direct incorporations, 5% indirect incorporations	36.9% of participants
Nefjodov et al. (2016) [62]	13 students (9 m, 4 f)	*Wii Fit* video game with a Wii Balance Board Duration: Two hours	✓			5.6% of dream reports, according to external ratings 19.4% of dream reports, according to subjective ratings	15.4% of participants, according to external ratings 54% of participants, according to subjective ratings
Wamsley et al. (2016) [63]	100 students (40 m, 60 f)	Virtual maze task Duration: 35 min.	✓	N1 N2		1.9% of dream reports	11.8% of participants
Fogel et al. (2018) [64]	24 healthy volunteers (4 m, 20 f)	Virtual maze task (resembling the video game *Team Fortress*) *Grand Slam Tennis* video game for Wii Duration: 30 min.		N1		N/S	N/S
Solomonova et al. (2018) [65]	40 volunteers (meditators and nonmeditators; 20 m, 20 f)	*Wii Fit* video game (*Balance Bubble*) with a Wii Balance Board Duration: 7 min.	✓	N1 N2		19.1% of dream reports 25% of REM reports 17.5% of N1 reports 18.8% of N2 reports	15% of participants
Wamsley & Stickgold (2019) [66]	39 students (13 m, 26 f)	Virtual maze task Duration: 35 min.	✓	N1 N2		6.4% of dream reports 12.5% of morning REM reports 7.1% of N1 reports 2.1% of N2 reports 12.5% of morning N2 reports	47.1% of participants
Klepel & Schredl (2019) [67]	22 students (5 m, 17 f)	Sequence from the film *Four Rooms,* without sound Duration: 5 min.	✓	✓		N/S	N/S
**Home-Based Studies**							
Powell et al. (1995) [68]	20 volunteers, mostly students (10 m, 10 f)	Film depicting the ceremonial slaughter of a buffalo Duration: 30 min.			✓	N/S	47.4% of participants
Davidson et al. (2005) [69]	24 volunteers, mostly students (8 m, 16 f)	9/11 media coverage video Introductory psychology lecture video Duration: 20 min. each			✓	N/S	N/S
Wamsley et al. (2010b) [46]	43 students (16 m, 27 f)	*Alpine Race II*, a visuomotor skiing video game Duration: 45 min.		N1 N2		29.5% of mentation reports Among those, 23.6% contained imagery, 6% contained thoughts; 23.3% contained direct incorporations; 6.2% contained indirect incorporations 1.28% of morning dream reports 47% of mentation reports on the first post-stimulus night	65% of participants (on the first post-stimulus night)
Gackenbach et al. (2011) [70]	40 gamer students (34 m, 6 f)	*Mirror’s Edge* video game Duration: 26 min.			✓	N/S	N/S
Davidson & Lynch (2012) [71]	75 students (10 m, 65 f)	9/11 media coverage video Introductory psychology lecture video			✓	N/S	N/S
Solomonova et al. (2015) [72]	26 healthy volunteers (10 m, 16 f)	VR maze task Duration: 23 min.			✓	11% of dream reports (on the first post-stimulus day)	61.5% of participants
Flockhart & Gackenbach (2017) [73]	76 male students (high-end gamers and low-end gamers)	Fearful film sequence from *Misery* *Far Cry 3*, a combat video game *Minecraft*, a creative video game Scholarly search task on the computer Duration: 10 min.			✓	N/S	N/S
Gott et al. (2021) [74]	39 students (10 m, 29 f)	VR-assisted training of lucid dreaming, with dream-like video games Duration: 9 h in total over 12 sessions			✓	N/S	N/S
Ribeiro et al. (2021) [75]	57 students (13 m, 44 f)	VR spatial memory task			✓	35.3% of dream reports	22.2% of participants
**Laboratory and Home-Based Studies**							
Picard-Deland et al. (2020) [76]	137 volunteers, mostly students (52 m, 84 f)	VR flying task Duration: 15 min.	✓	N2 N3		7.1% of dream reports (flying dreams) collected in the laboratory 3.1% of REM reports 20% of NREM reports 10.6% of dream reports (flying dreams) on the first post-laboratory night 4.1% of dream reports (flying dreams) across all post-laboratory nights	4.4% of participants (at the laboratory) 22.1% of participants (across all post-laboratory nights)

Note. Laboratory-based studies involved within-laboratory dream collection via scheduled awakenings. Home-based studies involved dream collection at home. Laboratory and home-based studies involved both within-laboratory and at-home dream collection. N/S = Not specified. ^a^ The incorporation rate (% of dream reports) was determined by dividing the number of dream reports containing stimulus-related content by the total number of content-filled dream reports. Content-filled dream reports were defined as those arising from awakenings that resulted in dream recall and contained at least some content. The incorporation rate for specific sleep stages was computed by dividing the number of stimulus-related dream reports from a specific sleep stage by the total number of content-filled reports obtained from that same stage. ^b^ The incorporation rate (% participants) was calculated by dividing the number of participants who incorporated the stimulus in at least one dream report by the total number of participants who underwent the appropriate experimental treatment.

## Data Availability

Not applicable.

## Appendix A

Search strategy updated on 10 July 2023.

### Appendix A.1. PubMed

(“Motion Pictures” [MeSH Terms] OR “Television” [MeSH Terms] OR “Virtual Reality” [MeSH Terms] OR “Augmented Reality” [MeSH Terms] OR “film*” [Text Word] OR “movie*” [Text Word] OR “cinema*” [Text Word] OR “video*” [Text Word] OR “motion picture*” [Text Word] OR “moving picture*” [Text Word] OR “documentar*” [Text Word] OR “Television” [Text Word] OR “TV” [Text Word] OR “Virtual Reality” [Text Word] OR “VR” [Text Word] OR “Augmented Reality” [Text Word]) AND (“Dreams” [MeSH Terms] OR “dream*” [Text Word] OR “sleep mentation” [Text Word]).

n = 398 results.

### Appendix A.2. APA PsycNet (PsycInfo and PsycArticles)

((IndexTermsFilt: (“Films”) OR IndexTermsFilt: (“Television”) OR IndexTermsFilt: (“Television Viewing”) OR IndexTermsFilt: (“Virtual Reality”) OR IndexTermsFilt: (“Augmented Reality”)) OR (Any Field: (film*)) OR (Any Field: (movie*)) OR (Any Field: (cinema*)) OR (Any Field: (video*)) OR (Any Field: (“motion picture*”)) OR (Any Field: (“moving picture*”)) OR (Any Field: (documentar*)) OR (Any Field: (television)) OR (Any Field: (TV)) OR (Any Field: (“virtual reality”)) OR (Any Field: (VR)) OR (Any Field: (“augmented reality”))) AND ((IndexTermsFilt: (“Dreaming”) OR IndexTermsFilt: (“Dream Content”) OR IndexTermsFilt: (“Dream Recall”) OR IndexTermsFilt: (“REM Dreams”)) OR (Any Field: (dream*)) OR (Any Field: (“sleep mentation”))).

n = 1123 results (*PsycInfo* = 1064; *PsycArticles* = 57).

### Appendix A.3. Web of Science Core Collection

ALL = (film* OR movie* OR cinema* OR video* OR “motion picture*” OR “moving picture*” OR documentar* OR television OR TV OR “virtual reality” OR VR OR “augmented reality”) AND TS = (dream* OR “sleep mentation”).

n = 2831 results.
